# On the Therapeutic Action and Uses of Ergot of Rye

**Published:** 1844-11

**Authors:** 


### PRACTICAL MEDICINE, &c.

*On the Therapeutic Action and Uses of Ergot of Rye.*—An abstract of a paper upon this subject, by M. Sachero, Professor of Clinical Medicine in the University of Turin, is to be found in the American Journal for October, taken from the foreign press. We have not room for the whole abstract, but give the conclusions, with a few of the remarks.

Ergot has been found useful in hemorrhages from the uterus, epistaxis, pulmonary hemorrhage, and hæmaturia. Speirani cites two cases of abundant hemoptysis cured by it. "It has also been used by Bazzoni, who, in 1831, published a work on the subject, in which he announced the following conclusions: 1st,

That the ergot of rye is a certain remedy in uterine hemorrhage and leucorrhœa. 2d, That the disagreeable sensations caused by it in the head are merely temporary. 3d, That if administered with prudence, it is without danger. 4th, That is equally efficacious, whether the discharge be active or passive. 5th, That its use is beneficial, even in those cases where the uterus and its appendages are affected with organic disease. 6th, That menstruation is not disturbed by its use."

M. Sachero has used it in cases of involuntary seminal emission, and assures us that his success was invariable.

"He cites some cases of obstinate bronchitis, from his clinical reports, which yielded as by enchantment to the use of this remedy; and, lastly, a case of severe otorrhœa which occurred in a young lady of a lymphatic temperament, who, after angina, was attacked with suppurating otitis, accompanied with head symptoms and a fever. Repeated blood-letting and the other usual remedies were tried, without the least avail. Injections into tne ear were then tried, consisting of an infusion of the ergot, made with 4 grammes of the latter to 120 grammes of boiling water; the medicine was, at the same time given internally; there was immediate melioration, and a complete cure followed in the course of a month."

The Pharmaceutical researches of M. Bonjean contained in the paper, inform us, that the active principles are reduced to two, the watery extract, soluble in water; and the resinous extract, soluble in alcohol. They are prepared by treating the powdered ergot with boiling water, in the displacement apparatus. The strong infusion, upon cooling, deposites the resinous extract, which may be purified by re-solution in alcohol. The oil floats upon the infusion, and may be separated by decantation. The watery extract is then obtained by evaporation.

The astringent (hemostatic) power of the ergot, appears to reside in the watery extract, which is further thought rather to retard than hasten labor. The power of exciting uterine contractions, is supposed to reside in the resinous extract. To hasten labor, the use of the powdered ergot without preparation, is considered the most favorable. The whole of the poisonous principle of the ergot is thought to reside in the oil.

"*Conclusions..*—From what precedes, then, it follows, 1st. That the watery extract, (hemostatic extract or ergotine of Bonjean,) is a hyposthenic remedy acting on the general vascular system; and that by means of it we can control hemorrhage, morbid sero-mucous discharges, and lessen over-action of the heart. Its action is clearly demonstrated to be on the vessels of the uterus, because, by its aid, we can control menorrhagia, threatened abortion, slight metritis and excitement of the uterine capillaries. The circulatory system being dependant on the great intercostal nerve, it follows that the action of the watery extract extends to this nerve and its numerous ramifications, as especially to those which preside over the life and functions of the uterine vessels. 2d. The resinous extract probably acts as a stimulant and its action extends to the nerves both of sensation and motion of the uterus. It is highly probable that when the ergot is administered in powder, it is in this extract that the principle resides which rouses into activity, the inert uterine contractions which had previously commenced. 3d. The action of the ergot, when administered in its natural state, appears to be of two kinds; the one, as in labor, affects the sanguineous system, the energy of which it diminishes (hyposthenises) by means of the ergotine; the other is upon the nerves of the uterus, which it stimulates by its resinous principle. To this double action must be added a third, equally hyposthenic, that of the oily or poisonous principle. Thus, then, in practice, several indications may be fulfilled by the isolated administration of these principles, and by the ergot in its natural state. The study of these cannot fail to extend its power as a therapeutic agent, when we have first determined the special circumstances in which they should be applied. 4th. The ergot only acts beneficially in labor, if this process has already commenced, when the amnion is ruptured, the position of the child natural, and the uterine contractions have been arrested or enfeebled, either by oppression of the forces, or by actual debility. In this latter case, the resinous extract is to be preferred to the watery or ergotine, consequently, the ergot has no effect in inducing abortion or labor, unless there is previously a commencement of uterine action. There is, nevertheless, an exception to this rule; and that is, when the fœtus is dead, or the uterus contains a tumor; but when this occurs, the uterus is in an unhealthy state, and, most generally, the ergot only acts by exciting the organ to contract, or facilitates and hastens the operation if already begun. If the ergot is given in large and repeated doses, previous to the commencement of labor, it either destroys the child, producing immediate labor, or at all events it sickens it. 5th. Its use is strongly indicated in hemorrhage, arising from a partial detachment of the placenta. In this case, life, as is well known, is in danger, if the flooding is great, and labor not speedily accomplished. The ergot, in its natural state, or one or other of the extracts, may be prescribed, according to the state of the patient. There are cases where the woman suffers from a true and general plethora of the uterus, recognizable by the state of the pulse, which is full and slow, dyspnœa, the swollen state of the veins of the hands, legs and feet, which become blue, and by a severe throbbing headache. In these cases, the patient should be bled once or twice, and then, if the uterus still continue inert, the ergot in its natural state may be prescribed, if it is thought proper and necessary to excite labor; if this is not considered desirable, then the ergotine should be had recourse to, if we wish to prevent hemorrhage and abortion. A bleeding should always precede the remedy in cases of congestion of the uterus. 6th. If the uterus does not expel the placenta, spontaneously, within a few hours after the birth of the child, the use of the ergot re-exerts the contractions in the course of seven or eight minutes, or in a quarter of an hour at most. 7th. In the preparation of the remedy it is an essential circumstance that the ergot be not gathered till it has reached a state of perfect maturity, toward the end of harvest, and in places with a free eastern exposure. If it be not perfectly ripe, it has either no action, or it is very feeble, as shown by the experiments of Bonjean. In this case it merely contains a little watery extract or ergotine, but no fixed oil, and consequently, is not poisonous. It is probable that it has been owing to the different degrees of maturity of the ergot, that the different effects, observed by certain authors, are to be attributed. It is also known that the ergot loses its virtue if it has been gathered more than a year, or if it is worm-eaten, has been exposed to the air, been roasted at too high a temperature, &c. It should only be reduced to powder when about to be used. 8th. It is more advantageous to give the ergot in small and repeated doses than in large ones which are often rejected by the stomach. An agreeable way of giving it is to suspend it in mucilage and add some aromatic syrup. We have already spoken of the manner of preparing an infusion by boiling water; the oil may be separated by decantation, and there remains the pure ergotine. The infusion may also be made with cold water. The decoction allowed to cool is little more than an infusion made in the warm way.

"This interesting article concludes with several cases of uterine hemorrhage cured by means of the watery extract; and with a case of abundant sero-mucuous discharge from the genital organs, in a girl three years of age, which had resisted all the ordinary means of treatment, but was cured by a single dose, (60 centigrammes*) of the powder infused in warm water, and then allowed to cool.—*Lond. and Ed. Month. Journ. Med. Sci., Aug.* 1844, from *Giornalle delle Scienze Mediche della Societa Medico-Chirurgica di Turino*, in the *Annales de Therapeutique.*

